# Is possible to rule out clinically significant prostate cancer using PI-RADS v2 for the assessment of prostate MRI?

**DOI:** 10.1590/S1677-5538.IBJU.2018.0382

**Published:** 2019-09-02

**Authors:** Publio Cesar Cavalcanti Viana, Natally Horvat, Valter Ribeiro dos Santos, Thais Carneiro Lima, Davi dos Santos Romão, Luciana Mendes de Oliveira Cerri, Marilia Germanos de Castro, Herbert Alberto Vargas, Júlia Azevedo Miranda, Claudia da Costa Leite, Giovanni Guido Cerri

**Affiliations:** 1 Departamento de Radiologia do Hospital Sírio-Libanês, São Paulo, SP, Brasil;; 2 Departamento de Patologia do Hospital Sírio-Libanês, São Paulo, SP, Brasil; 3 Departamento de Radiologia, Memorial Sloan Kettering Cancer Center, Nova York, NY, EUA

**Keywords:** Prostate, Magnetic Resonance Imaging, Neoplasms, Prostatic Neoplasms

## Abstract

**Objectives:**

To evaluate the diagnostic performance and interobserver agreement of PI-RADS v2.

**Materials and Methods:**

In this Institutional Review Board approved single-center retrospective study, 98 patients with clinically suspected PCa who underwent 3-T multiparametric MRI followed by MRI/TRUS fusion-guided prostate biopsy were included from June 2013 to February 2015. Two radiologists (R1 and R2) with 8 and 1 years of experience in abdominal radiology reviewed the MRI scans and assigned PI-RADS v2 scores in all prostate zones. PI-RADS v2 were compared to MRI/TRUS fusion-guided biopsy results, which were classified as negative, PCa, and significant PCa (sPCa).

**Results:**

Sensitivity, specificity, NPV, PPV and accuracy for PCa was 85.7% (same for all metrics) for R1 and 81.6%, 79.6%, 81.2%, 80.0% and 80.6% for R2. For detecting sPCa, the corresponding values were 95.3%, 85.4%, 95.9%, 83.7% and 89.8% for R1 and 93.0%, 81.8%, 93.7%, 86.7% and 86.7% for R2. There was substantial interobserver agreement in assigning PI-RADS v2 score as negative (1, 2, 3) or positive (4, 5) (Kappa=0.78). On multivariate analysis, PI-RADS v2 (p <0.001) was the only independent predictor of sPCa compared with age, abnormal DRE, prostate volume, PSA and PSA density.

**Conclusions:**

Our study population demonstrated that PI-RADS v2 had high diagnostic accuracy, substantial interobserver agreement, and it was the only independent predictor of sPCa.

## INTRODUCTION

Prostate cancer (PCa) is one of the most common cancer, being the most commonly diagnosed cancer in men in the United States and the second most common one worldwide (1). The detection of clinically significant PCa (sPCa) is gathering growing interest in the literature, because a significant number of patients with indolent tumor has been unnecessarily treated with aggressive treatment, with potential complications (2).

International PCa diagnosis and management guidelines are predominantly based on literature originating from developed countries (3-6). This is also the case for cancer imaging guidelines, such as the Prostate Imaging Reporting and Data System (PI-RADS) (7). The use of multiparametric prostate magnetic resonance imaging (mpMRI) has increased exponentially in the last few years in several countries, supported by vast literature demonstrating its usefulness in multiple contexts regarding PCa diagnosis, treatment planning and selection, determination of active surveillance eligibility and follow-up and post-treatment assessment. PI-RADS was originally introduced in 2012 (8) and updated as a version 2.0 in 2015 (7), with the primary aim of standardizing multiparametric prostate MRI acquisition and reporting. It is based on a combination of the existing literature synthesized by an expert panel.

There is increasing literature validating the use of PI-RADS v2 concerning accuracy and repeatability (9-13). In this scenario, the purpose of the study was to evaluate the diagnostic performance and interobserver agreement of PI-RADS v2 in detecting clinically significant PCa in a Brazilian population.

## MATERIALS AND METHODS

### Study population

In this single-center retrospective study, Institutional Review Board approval was obtained and the requirement for informed written consent was waived. The Hospital Sírio-Libanês database was retrospectively queried to identify patients who underwent 3-T mpMRI followed by MRI/TRUS fusion-guided prostate biopsy from June 2013 to February 2015.

Inclusion criteria were: (a) patients with clinically suspected PCa, based on increased PSA levels and/or abnormal digital rectal examination, and (b) MRI/TRUS fusion-guided prostate biopsy performed within 6 weeks following the date of the 3-T mpMRI. The exclusion criteria were patients with previous diagnosis of PCa, history of prostate biopsy up to 3 months before the MRI, or histological data unavailable for review.

We included 106 consecutive patients who underwent 3-T mpMRI followed by MRI/TRUS fusion-guided prostate biopsy within 6 weeks during the selected period. We excluded 8 patients: 5 patients with known PCa, 2 patients with recent prostate biopsy, and 1 patient due to absence of histological samples. All 98 remaining patients were included in the final study population. The median interval between the 3-T mpMRI and MRI/TRUS fusion-guided prostate biopsy was 14 days (range, 2-42).

### Prostate mpMRI

Prostate mpMRI was performed using a 3.0-T GE Signa HDxt MR Scanner (GE Healthcare, Milwaukee, USA) with receive only pelvic phased-array coil with 18 channels without endorectal coil. All patients fasted for at least 4 hours before the examination. The prostate images were acquired before and after intravenous injection of 0.2mL/kg of gadoversetamide (Optimark; Mallinckrodt Inc., St. Louis, MO) at a rate of 3mL/second by power injector, followed by 20mL of saline flush.

The MRI examinations were performed using a standardized clinical protocol as recommended in PI-RADS v2 (7), including T2-weighted imaging (T2WI), dynamic contrast-enhanced (DCE) imaging, and diffusion-weighted imaging (DWI) (Table-1).

**Table 1:** Sequence parameters of prostate mpMRI.

Parameter	Axial T2WI	Axial DWI (B50 / 1500 /2000)	Multi plane T2 3D	Axial Dixon Pelvis	Axial DCE
Repetition time (msec)	7500	5500	1200	5.73	3.80
Echo time (msec)	116	84	126	2.46 and 3.69	1.41
Flip angle (degrees)	150	-	120	90	150
Field of view (mm)	150	300	300	280	180
Acquisition matrix	218 x 256	144 x 160	256 x 256	192 x 256	154 x 192
Section thickness (mm), no gaps	3	3.5	1.17	3.0	3.5
Reconstruction voxel imaging resolution (mm/pixel)	0.3 x 0.3	1.5 x 1.5	0.59 x 0.59	1.1 x 1.1	0.9 x 0.9
Acquisition time (min:sec)	04:15	08:43	06:59	00:16	04:58

### Image analysis

The radiologists were blinded to clinical status, initial report, laboratory tests and histopathological results.

Both readers had been routinely using PI-RADS v2 as part of their clinical practice prior to this study. In addition, both readers met for one hour and reviewed the PI-RADS v2 literature and instructions together with 15mpMRI cases (not in the study population) to practice and align interpretation of the scoring system (7). In patients with more than 1 lesion, the index lesion (IL) was selected. We defined as IL the one with the highest PI-RADS score, when multiple lesions had the same score, the largest one, measured as the largest dimension on T2-weighted images in any plane. The ILs were described by location, dimensions (mm), and PI-RADS v2 score. Prostate mpMRI exams were considered positive if the PI-RADS scores were 4 or 5.

### Transrectal US-guided biopsy

All patients underwent MRI/TRUS fusion-guided prostate biopsy using an iU22 Ultrasound System (Philips, Amsterdam, Netherlands), with an operating bandwidth of 8-4MHz equipped with an end-fire endorectal biopsy probe. All biopsies were performed by a board-certificated interventional radiologist X.Z. with 20 years of experience and were supervised by the radiologist who had performed the prostate mpMRI during clinical routine. Standardized 12-core biopsy was performed and additional cores were taken from the suspicious areas in mpMRI using the image fusion approach. In this method, transrectal TRUS was performed by the radiologist and the MRI, which was performed previously, was fused with the real-time TRUS using a digital overlap. Therefore, the suspicious areas previously delineated on MRI were possible to be target on US. All prostatic cores were obtained by using an 18-gauge biopsy needle (Argon Medical Devices, Athens, Texas, USA) and were labeled to identify the location. The median number of biopsy cores per patient was 21 (IQR, 18-22). Of these, 12 standardized core biopsies and 6 to 10 were made by the MRI/TRUS fusion technique directed to the suspicious areas.

### Pathological analysis

Two genitourinary pathologists (X.X.Y and Y.Y.H.) with 20 and 10 years of experience reviewed the samples according to International Society of Urological Pathology Consensus (14), regarding the presence of tumor, if present, the Gleason score, the number of cores with tumor and the percentage of tumor in each core.

### Statistical analysis

We classified the PI-RADS v2 as negative (scores 1 to 3) or positive (scores 4 or 5) and final diagnosis, based on clinical status and biopsy results, as negative, any PCa, and significant sPCa (sPCa). sPCa was defined as those of non-very low risk group, according to risk classification adopted on National Comprehensive Cancer Network Guidelines (NCCN), including low, intermediate, high, and very high risk, and metastatic (3). Men with all of the following tumor characteristics are categorized in the very-low-risk group: clinical stage T1c, biopsy Gleason score ≤6/Grade Group-1, PSA <10ng/mL, presence of disease in fewer than 3 biopsy cores, ≤50% prostate cancer involvement in any core, and PSA density <0.15ng/mL/g. Data were analyzed through the statistical program Software SPSS 22.0 version, using chi-square and Mann-Whitney U tests. Multivariate logistic regression was also used if association were detected on univariate analysis. P values p <0.05 were considered significant. Interobserver agreement on PI-RADS v2 (grouped as scores 1-3 vs. 4-5) was assessed using weighted Kappa. Kappa values were interpreted as follows: 0.00-0.20, slight agreement; 0.21-0.40, fair agreement; 0.41-0.60, moderate agreement; 0.61-0.80, substantial agreement; and 0.81-1.00, almost perfect agreement (15).

## RESULTS

### Baseline demographics and prostate mpMRI findings

Considering the skewed distribution of our data we used median and IQR to demonstrate our results. There were 98 patients with a median age of 60 years (IQR: 54-69), median serum PSA of 6.3 ng/mL (IQR: 4.5-9.7), median PSA density of 0.15ng/mL/g (IQR: 0.09-0.23) and median prostate volume of 32cm^3^ (IQR: 40.5-56.8). Twenty-one of the 98 patients (21.4%) had an abnormal digital rectal examination.

The mean size of the index lesion on mpMRI was 14.4mm (R1=14.3mm; R2=14.5mm). According to R1 and R2, 49/98 (50%) and 50/98 (51%) of patients were assigned PI-RADS scores of 4 or 5. Forty-nine of 98 (50%) men were diagnosed with PCa and 43/98 (44%) with sPCa, 84% were located in the peripheral zone and 16% in the transition zone, for both PCa and sPCa combined. Among the 49 patients with PCa, 11 (22%) were assigned a Gleason score of 6 and 38 (78%) the Gleason scores were greater than or equal to 7 (median, 7; IQR: 7-8). The characteristics of the patients without PCa, with PCa and sPCa are displayed in Table-2, the whole distribution of Gleason score (6, 7, 8, 9 and 10) in groups is displayed in Table-3, the distribution of PIRADS classification in each category is displayed in Table-4, the accuracies, sensitivities, specificities, negative predictive values, and positive predictive values of PI-RADS v2 for the diagnosis of PCa and SPCa are displayed in Table-5 and the numbers that allow estimation of the different diagnostic accuracy parameters are displayed in Table-5.1.

**Table 2:** Characteristics of the 43 patients with sPCa. ª

Parameter	% (n/total)
DRE abnormal	34.8% (15/43)
Gleason score > 6	88.4% (38/43)
PSA > 10 ng/mL	55.8% (24/43)
PSA density ≥ 0.15 ng/mL/g	72.1% (31/43)
Three or greater than 3 prostate biopsy cores positives	90.7% (39/43)
> 50% cancer in any biopsy core	83.7% (36/43)

^a^ Clinically significant prostate cancer was considered as those non-very low risk group as according to National Comprehensive Cancer Network (NCCN).

**Table 3:** Distribution of Gleason score (6, 7, 8, 9 and 10) in groups for patients with sPCa. ª

Parameters	% (n/total)
Gleason 6	5/43 (11.6%)
Gleason 7	19/43 (44.1%)
Gleason 8	10/43 (23.2%)
Gleason 9	9/43 (20.9%)

ª Clinically significant prostate cancer was considered as those non-very low risk group as according to National Comprehensive Cancer Network (NCCN).

**Table 4:** Distribution of PIRADS classification in each category.

	WITHOUT PCa			TOTAL CANCER			sPCa			PCa	
	RAD 1	RAD 2	RAD 1		RAD 2	RAD 1		RAD 2	RAD 1		RAD 2
PI-RADS 1 or 2	28/49 (57.1%)	32/49 (65.3%)	3/49 (6.1%)		6/49 (12.2%)	0/43 (0%)		1/43 (2.3%)	3/6 (50%)		5/6 (83.3%)
PI-RADS 3	13/49 (26.5%)	7/49 (14.2%)	4/49 (8.1%)		3/49 (6.1%)	2/43 (4.6%)		2/43 (4.6%)	2/6 (33.3%)		1/6 (16.6%)
PI-RADS 4	5/49 (10.2%)	10/49 (20.4%)	23/49 (46.9%)		18/49 (36.7%)	22/43 (51.1%)		18/43 (41.8%)	1/6 (16.6%)		0/6 (0%)
PI-RADS 5	2/49 (4%)	0/49 (0%)	19/49 (38.7%)		22/49 (44.8%)	19/43 (44.1%)		22/43 (51.1%)	0/6 (0%)		0/6 (0%)

	WITHOUT PCa			TOTAL CANCER			sPCa			PCa	
	RAD 1	RAD 2	RAD 1		RAD 2	RAD 1		RAD 2	RAD 1		RAD 2

PI-RADS 1, 2 or 3	42/49 (85.7%)	39/49 (79.6%)	7/49 (14.3%)		9/49 (18.4%)	2/43 (4.7%)		3/43 (7%)	5/6 (83.3%)		6/6 (100%)
PI-RADS 4 or 5	7/49 (14.3%)	10/49 (20.4%)	42/49 (85.7%)		40/49 (81.6%)	41/43 (95.3%)		40/43 (93%)	1/6 (16.6%)		0/6 (0%)

**Table 5:** Accuracies, sensitivities, specificities, negative predictive values, and positive predictive values of PI-RADSv2 for the diagnosis of PCa and SPCa.

	RADIOLOGIST 1		RADIOLOGIST 2	
	
	PCa % (CI)	sPCa % (CI)	PCa % (CI)	sPCa % (CI)
**Accuracy**	85.7% (77.4–91.3)	89.8% (82.2 – 94.4)	80.6% (71.7–87.2)	86.7% (78.6–92.1)
**Sensitivity**	85.7% (73.3–92.9)	95.4% (84.5 – 98.7)	81.6% (68.6–90.0)	93.0% (81.4–97.6)
**Specificity**	85.7% (73.3–92.9)	85.4% (73.8 – 92.4)	79.6% (66.4–88.5)	81.8% (69.7–89.8)
**NPV**	85.7% (73.3–92.9)	95.9% (86.3 – 98.9)	81.2% (68.1–89.8)	93.7% (83.2–97.8)
**PPV**	85.7% (73.3–92.9)	83.7% (71.0 – 91.5)	80.0% (67.0–88.8)	86.7% (78.6–92.1)

**PZ** = peripheral zone; **TZ** = transitional zone; **NPV** = negative predictive value; **PPV** = predictive positive value

**Table 5.1:** Numbers that allow estimation of the different diagnostic accuracy parameters.

	Patients with sPCa	Patients without sPCa	p-value
	
	Frequency (%)	Frequency (%)	
**PIRADS (RAD 1)**			
Positive (4 or 5)	41 (95.3%)	8 (14.5%)	<0.001
Negative (1,2 or 3)	2 (4.7%)	47 (85.5%)	
**PIRADS (RAD 2)**			
Positive (4 or 5)	40 (93.0%)	10 (18.2%)	<0.001
Negative (1, 2 or 3)	3 (7.0%)	45 (81.8%)	

	Patients with PCa	Patients without PCa	p-value
	
	Frequency (%)	Frequency (%)	

**PIRADS (RAD 1)**			
Positive (4 or 5)	42 (85.7%)	7 (14.3%)	<0.001
Negative (1, 2 or 3)	7 (14.3%)	42 (85.7%)	
**PIRADS (RAD 2)**			
Positive (4 or 5)	40 (8.6%)	10 (20.4%)	<0.001
Negative (1,2 or 3)	9 (18.4%)	39 (79.6%)	

### Diagnostic performance of prostate mpMRI

For the detection of PCa, R1 had the same sensitivity, specificity, NPV, PPV and accuracy (85.7%). The sensitivity and specificity of R1 for the detection of sPCa were 95.3% and 85.4%, and the NPV and PPV were 95.9% and 83.7%, respectively. The overall accuracy for detection of sPCa was 89.8%.

For R2, the sensitivity, specificity, NPN and PPV for detection of PCa were 81.6%, 79.6%, 81.2%, and 80.0%, respectively and the accuracy was 80.6%. Considering the sPCa, the sensitivity and specificity were 93.0% and 81.8%, the NPV and PPV were 93.7% and 86.7% and the accuracy was 86.7%. The performances of readers separately are summarized in Table-3.

The interobserver agreement in assigning PI-RADS v2 score was substantial (Kappa=0.78).

### sPCa missed on prostate mpMRI

**Figure 1:**
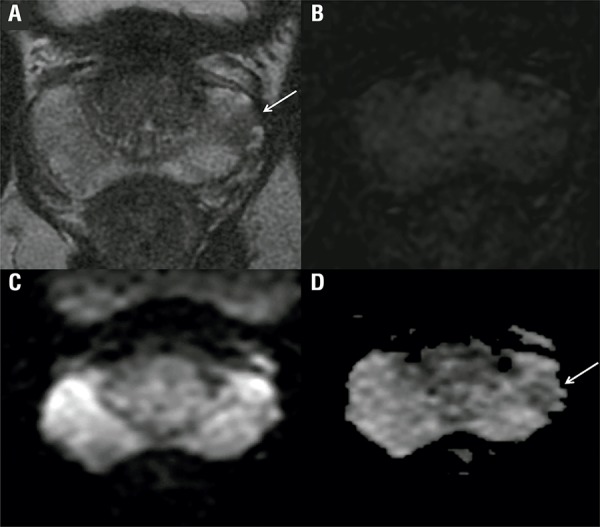
A focal lesion was identified by both radiologists on T2WI (a) in the prostate left middle third, but with slightly restricted diffusion (c, d) and negative perfusion (b). PI-RADS final score 3 was assigned by the two readers. Pathological analysis revealed Gleason 8 in the right middle third and Gleason 7 in the left.

**Figure 2:**
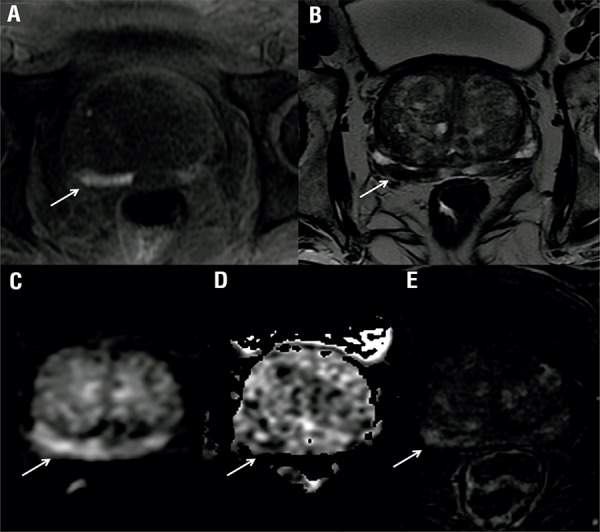
MpMRI showing suspected lesion on T1WI (a), T2WI (b), DWI (c), ADC map (d) and perfusion (e) sequences. The radiologist 1 identified lesion in the prostate right middle third and assigned as PI-RADS 5 with extra-prostatic extension. The same lesion was scored as 2 for the radiologist 2. The pathological analysis diagnosed a Gleason 6 in the right middle third. The patient was classified as having significant cancer due to PSA of 10ng/mL. In this case, the presence of blood may have limited the interpretation of the reader 2.

R1 missed 2 patients with sPCa, one with PSA density >0.15ng/mL/g and one with Gleason of 8 (4+4). R2 missed the same 2 cases in addition to another case with serum PSA of 10ng/mL and more than 3 prostate biopsy cores positive and 90% cancer in any core. All sPCa missed were located in the peripheral zone. Figure-1 illustrates one representative false negative case. Figure-2 demonstrates a case of disagreement between the readers.

### Clinical risk stratification

Serum PSA, PSA density, prostate volume and PI-RADS v2 scores were associated with sPCa on univariate analysis (p <0.05 in each). However, on multivariate analysis, PI-RADS v2 was the only significant independent predictor of sPCa with an odds ratio of 120 (95% CI: 24-599, p <0.001) for R1 and odds ratio of 60 (95% CI: 15-233, p <0.001) for R2.

## DISCUSSION

In our study cohort of 98 Brazilian patients with clinically suspected PCa, we found accuracy for both readers in detecting sPCa on mpMRI using PI-RADS v2 higher than 86% and a NPV higher than 93%. Our study also demonstrates a substantial interobserver agreement in assessing the PI-RADS v2 score as negative or positive. Furthermore, PI-RADS v2 was the only independent predictor of sPCa on multivariate analysis.

Using PI-RADS v2 it was possible to rule out the vast majority of sPCa with substantial reproducibility between 2 independent radiologists, even broadening the criteria of significant tumors as we did including low risk patients as sPCa (3). The reason that motivated us to include it was the fact that our gold standard was transrectal biopsy, and it is known that there is a potential risk of patients classified as low risk PCa on biopsy to end up with sPCa after radical prostatectomy (16, 17). On the other hand, in contrast to other studies, on multivariate analysis PSA density was not significantly correlated with sPCa, probably due to our small sample size.

With regards to accuracy of PI-RADS v2, our results are consistent with prior studies, which have reported accuracies ranging from 70% to 87% (9, 18). Our results are equivalent even if we compare it with studies in which the endorectal coil was used (9). These reinforce the notion that 3T mpMRI with pelvic phased-array coil is comparable to 1.5TmpMRI for detection of PCa (19).

The interobserver agreement in assigning the PI-RADS v2 varies in the literature from moderate to substantial (9-11, 18), being the moderate more frequent, even when selected key-images were used in these studies. Our substantial interobserver agreement (kappa=0.78) is comparable with that demonstrated by Kasel-Seibert et al. (kappa=0.68) (10), which also evaluated the interobserver agreement between radiologists from the same institution. Nevertheless, it was better than the results demonstrated by Muller et al. (kappa=0.46) (9) and Rosenkrantz et al. (kappa=0.59 in peripheral zone; and kappa=0.51 in transition zone) (11) among readers from different centers.

In the other study from Brazil, 54 patients were also retrospectively included and 2 readers with different levels of experience in uroradiology (1 and 10 years) reviewed the mpMRI. The primary outcome was the histological analysis after biopsy or surgery, but without classification between PCa and sPCa. In comparison to this study, our results of diagnostic performance of mpMRI in the diagnosis of PCa were overall similar; however, our values of NPV were higher (81%-86% vs. 66%-67%) whereas our PPV results were slightly lower (80% to 86% vs. 87% to 89%). With regards to interobserver agreement, our result was better than those presented by them (kappa=0.78 vs. kappa=0.53), but it was not specified if the analysis was made considering each score separately or grouped in negative vs positive (18).

Our observations suggest that PI-RADS v2 is feasible to be implemented in institutions without previous experience on that, and it is reproducible between readers with different expertise after a specific training. Our results are similar with those performed in different study populations such as in the US and Europe, which emphasize the added value of PI-RADS v2 to standardize the acquisition, interpretation, and reporting of prostate mpMRI. Using PI-RADS v2 it was possible to rule out the vast majority of sPCa, however, the fact that the readers did not detect some patients with sPCa reinforces that the approach of PCa should not be focus on a sole exam, but on a multidisciplinary approach, which includes the mpMRI.

There are several limitations to our study. First, it was a retrospective assessment with inherent limitations of this study design and small sample size. In addition, the gold standard was transrectal MR/ultrasound fusion biopsy, which is known to potentially underestimate the diagnosis and grade of the PCa, on the other hand it reflects the reality of the clinical management of patients with suspected PCa, who don’t all proceed to surgery which provides the best standard of reference (whole-mount prostatectomy specimen). Considering that, our results may be overestimated. Furthermore, considering that we did not use whole-mount as the reference standard it was not possible to do a precise correlation between mpMRI and pathology. Additionally, even though the readers have different levels of expertise, they work at the same institution which could have overestimated the inter-observer agreement; however, they had different educational background. Finally, considering that the study was performed at a comprehensive cancer hospital, we may have included a high proportion of significant PCa when compared with other center, it also could have overestimate the performance of the PI-RADS and of the readers.

In conclusion, our study in a Brazilian population demonstrates high diagnostic accuracy of PI-RADS, and also a substantial interobserver agreement in differentiating PI-RADS v2 1, 2 and 3 from PI-RADS 4 and 5 between readers with different levels of expertise. Overall, the mpMRI using PI-RADS v2 could rule out the vast majority of sPCa and it was the only independent predictor of sPCa.
